# Antinociceptive effect of some extracts from *Ajuga chamaecistus* Ging. ssp*. tomentella* (Boiss.) Rech. f. aerial parts

**DOI:** 10.1186/2008-2231-22-56

**Published:** 2014-07-14

**Authors:** Mahnaz Khanavi, Araz Mohammad Davoodipoor, Seyede Nargess Sadati, Mohammad Reza Shams Ardekani, Mohammad Sharifzadeh

**Affiliations:** 1 Department of Pharmacognosy and Medicinal Plants Research Center, Faculty of Pharmacy, Tehran University of Medical Sciences, Tehran 14155-6451, Iran; 2 Department of Traditional Pharmacy, School of Traditional Iranian Medicine and Traditional Iranian Medicine and Pharmacy Research Center, Tehran University of Medical Sciences, Tehran, Iran; 3 Department of Pharmacology and Toxicology, Faculty of Pharmacy, Tehran University of Medical Sciences, Tehran, P.O. Box 14155–6451, Iran

**Keywords:** *Ajuga chamaecistus* ssp. *tomentella*, Antinociceptive effect, Analgesic activity, Formalin test, Mice

## Abstract

**Background:**

The genus *Ajuga* is used for the treatment of joint pain, gout, and jaundice in traditional Iranian medicine (TIM). *Ajuga chamaecistus* ssp. *tomentella* is an exclusive subspecies of *Ajuga chamaecistus* in the flora of Iran. The aim of this study was to evaluate antinociceptive properties of some extracts from aerial parts of *A. chamaecistus* ssp*. tomentella.*

**Methods:**

Antinociceptive activities of total water and 80% methanol extracts, hexane, diethyl ether and n-butanolic partition fractions of the methanolic extract were analyzed using the formalin test in mice. Indomethacin (10 mg/kg) and normal saline were employed as positive and negative controls, respectively.

**Results:**

Oral administration of all extracts (200, 400 and 600 mg/kg) 30 min before formalin injection had no effect against the acute phase (0–5 min after formalin injection) of the formalin-induced licking time, but hexane fraction (200 mg/kg) caused a significant effect (p < 0.001) on the chronic phase (15–60 min after formalin injection). Total water and diethyl ether extracts at a dose of 400 mg/kg showed a very significant analgesic activity on the chronic phase (p < 0.001 and p < 0.01, respectively).

**Conclusions:**

The results of this study suggest that the extracts of *A. chamaecistus* ssp*. tomentella* have an analgesic property that supports traditional use of *Ajuga* genus for joint pain and other inflammatory diseases.

## Background

Five species of genus *Ajuga* (Lamiaceae) are found in the flora of Iran in which *Ajuga chamaecistus* has been contained several endemic subspecies including *A. chamaecistus* ssp. *tomentella*[1]. Some species which belong to this annual and perennial genus are used as the medicinal plant in the traditional medicine of several countries mostly in Africa, Asia, and China as for wound healing; anthelmintic, antifungal, antifebrile, antitumor, antimicrobial, and diuretic agent, and for the treatment of hypertension, hyperglycemia, joint pain, etc. [2-4]. *Ajuga chamaepitys* (L.) Schreb. which grows in the Middle East and Asia has been used in the treatment of rheumatism, gout, dropsy, jaundice, and sclerosis. *A. decombens* Thunb. that originally grows in East Asia is used for analgesia, inflammation, fever, and joint pain [5]. Moreover in Iranian traditional medicine, the genus *Ajuga* (Kamaphytus) has been used for treatment of joint pain, gout, and jaundice [6].

Also, several biological studies have been performed on many species of this genus which have confirmed their ethno pharmacological properties such as hypoglycemic [7], anti-inflammatory [8], anabolic, analgesic, anti-arthritis, antipyretic, hepatoprotective, antibacterial, antifungal, antioxidant, cardiotonic [5], and antimalarial [9] properties and their application in the treatment of joint diseases [10].

As well, many phytochemical studies on *Ajuga* species have been performed which have led to the isolation of phytoecdysteroids [11,12], neo- clerodanediterpenoids [13], phenylethyl glycosides [3], withanolides [2], iridoids and flavonoids [14], and essential oils [15].

Prior to this study, we have isolated 10 compounds; 20-hydroxyecdysone, cyasterone, ajugalactone, makisterone A, and 24-dehydroprecyasterone (phytoecdysteroids), 8-acetylharpagide (iridoid), *cis*- and *trans*-melilotoside, lavandulifolioside, leonoside B, and martynoside (phenylethanoid glycosides), from diethyl ether and n-butanolic fractions of *Ajuga chamaecistus* ssp. *tomentella*. Cytotoxicity evaluation of some fractions of this plant showed the cytotoxicity of hexane fraction against normal and cancer cell lines. Most of the isolated compounds were inactive in the cytotoxicity assay [16,17].

The aim of this study was to evaluate antinociceptive effects of oral administration of total water and 80% methanolic extracts and partition fractions of hexane, diethyl ether and n- butanol obtained from methanolic extract of aerial parts of *Ajuga chamaecistus* ssp. *tomentella* in an attempt to validate the traditional use of the plants belonging to genus *Ajuga*.

## Methods

### Plant material

Aerial parts of *Ajuga chamaecistus* Ging. ssp. *tomentella* (Boiss.) Rech. f. were collected from “Sorkhe Hesar”, east of Tehran, Iran, in June 2008 and verified by Prof. G. Amin. A voucher specimen (THE-6697) was deposited in the herbarium of the Department of Pharmacognosy, Faculty of Pharmacy, Tehran University of Medical Sciences, Tehran, Iran.

#### Methanolic extraction

The air-dried and ground aerial parts of *A. chamaecistus* ssp. *tomentella* (250 g) were extracted with methanol 80% (3 × 0.5 L) at room temperature. The solvent was evaporated on a rotary evaporator and in a vacuum oven to give a dark brown extract (45 g). The extract (30 g) was suspended in 80% methanol and partitioned successively between 80% methanol, n-hexane, diethyl ether, and n-butanol. Removal of the solvents with a rotary evaporator resulted in the production of n-hexane, diethyl ether, and n-butanol fractions.

#### Water extraction

Two hundred and fifty grams of the powdered plant from the aerial parts of *A. chamaecistus* ssp. *tomentella* were extracted with distilled water (3 × 0.5 L) at room temperature. The solvent was removed with a rotary evaporator and freeze drying process to give an extract (30 g).

### Administration

The extracts were dissolved in normal saline to achieve the working concentrations. Extracts, standard drug (Indomethacin 10 mg/kg), and normal saline were administered by oral gavage. Three doses of 200, 400 and 600 mg/kg of all extracts were examined.

### Animals

Male albino mice weighing 25–30 g were obtained from Pasteur institute and housed in groups of 7 with a 12 h light–dark cycle and constant temperature (22°C). Mice were allowed to acclimatize to the laboratory for 30 min before the experiments began. This study was approved by the ethics committee of the Pharmaceutical Science Research Center of TUMS.

### Formalin test in mice

Twenty microliters of formalin (0.5%) was injected subcutaneously according to the previous study [18]. The total time (second) spent on licking in response to the injected paw in the acute phase (0–5 min) and chronic phase (15–60 min) after formalin injection was measured as a pain indicator.

### Statistical analysis

Results are expressed as mean ± standard error of mean (S.E.M). Statistical differences between the treatment and control groups were evaluated by one-way ANOVA, followed by Newman–Keuls post hoc test. p < 0.05 was considered significant.

## Results

**Figure 1:**
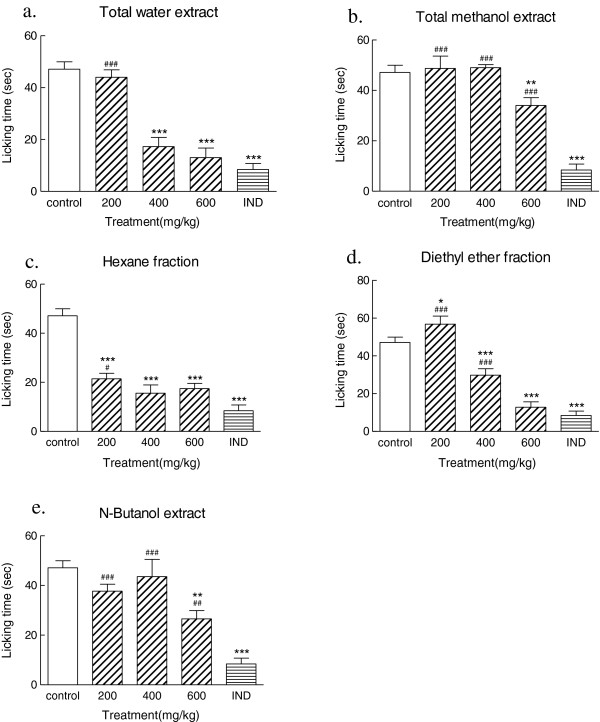
**Effects of different extracts of *Ajuga chamaecistus *ssp*. tomentella *on chronic phase of formalin- induced pain.** Different doses of all extracts (200, 400, 600 mg/kg), **a)** Total water extract, **b)** Total methanol extract, **c)** Hexane fraction, **d)** Diethyl ether fraction, and **e)** N-Butanol fraction, were administered to mice by oral tube. Control group received normal saline. All extracts of the plant were administered 30 min before formalin injection. Antinociception was recorded 15–60 min (Chronic phase), after formalin injection. Each point is the mean ± S.E.M of at least 7 animals. Control (normal saline), IND (Indomethacin). *p<0.05,** p < 0.01, ***p < 0.001 (as compared with normal saline), ^#^p<0.05, ^##^p < 0.01, ^###^p < 0.001 (as compared with Indomethacin).

**Figure 2:**
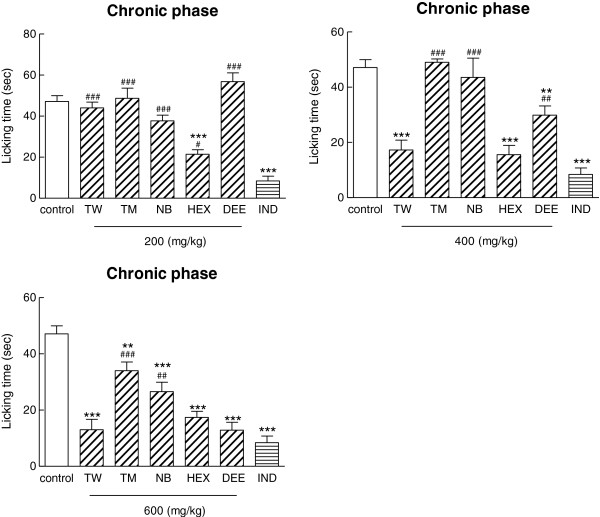
**Comparision of different treatments of *Ajuga chamaecistus *ssp*. tomentella *on chronic phase of formalin- induced pain.** All samples were administered to mice by oral tube. Control group received normal saline. All extracts of the plant were administered 30 min before formalin injection. Antinociception was recorded 15–60 min (Chronic phase), after formalin injection. Each point is the mean ± S.E.M of at least 7 animals. Control (normal saline), TW (total water), TM (total methanol), NB (n-butanol), HEX (hexane fraction), DEE (diethyl ether fraction). IND (Indomethacin). **p < 0.01, ***p < 0.001 (as compared with normal saline), ^#^p<0.05, ^##^p < 0.01, ^###^p < 0.001 (as compared with Indomethacin).

In the present study, antinociceptive activity of total water, 80% methanol extracts, and three fractions of methanolic extract from the aerial parts of *Ajuga chamaecistus* ssp. *tomentella* were evaluated in a nociception model in mice. All extracts were administered by a gastrointestinal tube at different doses (200, 400, and 600 mg/kg) 30 min before intraplantar injection of formalin (20 μl, 0.5%). The licking time was 40.29 ± 5.76 for indomethacin (10 mg/kg) in the acute phase. There was no significant difference between indomethacin and all treated extracts (Table 1).Figure 1 shows the antinociceptive effects of all extracts at different doses on the chronic phase of pain induction. Results demonstrated that hexane fraction (200 mg/kg), diethyl ether fraction and total water extract at a dose of 400 mg/kg significantly (p < 0.001) affected the duration of licking in the chronic phase, which was comparable to indomethacin (10 mg/kg). The total methanol extract (600 mg/kg) and n-butanolic (600 mg/kg) fraction also exhibited a reduction on the duration of licking in the chronic phase (p < 0.01).Comparison of the antinociceptive activity of all extracts showed that the maximum inhibitory response was obtained with 200 mg/kg of hexane fraction. According to results, there was no significant difference between the analgesic effect of total water extract and hexane fraction at a dose of 400 mg/kg, diethyl ether fraction (600 mg/kg), n-butanol fraction (600 mg/kg), and indomethacin (10 mg/kg) as an NSAID (Figure 2).

**Table 1:** **Influence of *Ajuga chamaecistus *** **ssp** ***. tomentella *** **extracts on acute phase of formalin- induced pain in mice**

	**Licking time(sec)**^**a**^				
**Treatment(mg/kg)**^**b**^	**TW**	**TM**	**HEX**	**DEE**	**NB**
Control	52.14 ± 1.35^c^	52.66 ± 1.47	52.60 ± 1.80	53.00 ± 1.67	52.83 ± 1.38
200	49.00 ± 3.4	53.43 ± 5.35	46.14 ± 2.54	53.86 ± 3.20	48.71 ± 2.02
400	58.86 ± 4.41	59.29 ± 2.41	47.86 ± 3.29	57.43 ± 3.64	46.86 ± 3.46
600	36.00 ± 5.21	59.57 ± 2.04	50.86 ± 3.04	45.71 ± 1.82	47.57 ± 2.44

^a^The amount of time spent licking the injected paw recorded in 0–5 min (acute phase).

^b^Control: Normal saline, TW: Total Water extract, TM: Total Methanol extract, HEX: Hexane fraction, DEE: Diethyl Ether fraction, NB: N-Butanol fraction.

^c^Results are expressed as the mean ± S.E.M (n = 7).

## Discussion

The formalin test provides a moderate and continuous pain because of tissue injury in the animal, which is a better approach to clinical conditions than more traditional tests of nociception [19]. Subcutaneous injection of formalin induces two distinctive periods of response. The early phase is explained as a direct stimulation of nociceptive neurons, and the late phase occurs secondary to the inflammatory reactions [20,21]. Drugs which affect the central nervous system such as opioids inhibit both phases equally while peripherally acting drugs such as aspirin and indomethacin inhibit the late phase [22]. The early phase of response to the formalin test is insensitive to anti-inflammatory drugs [23]. Inflammation in the late phase is due to the release of chemical mediators, such as serotonin, histamine, bradykinin and prostaglandins and at least to some degree, the sensitization of central nociceptive neurons [24].

The results showed that hexane (200 mg/kg), diethyl ether (400 mg/kg), and n-butanol fractions (600 mg/kg) from the methanolic extract (80%) in addition to total water extract (400 mg/kg) significantly decreased the pain related to the late phase (inflammatory agents) of the formalin test. Since the analgesic properties of effective extracts were observed in late phase like NSAIDs, the antinociceptive activities of the extracts are apparently mediated by interactions with inflammatory mediators especially arachidonic acid metabolites. Recently, [9] reported that 70% ethanol extract of whole plants of *Ajuga bracteosa* showed a significant topical anti-inflammatory activity and a strong in vitro COX-1 and COX-2 inhibitory effect. Among the isolated compounds from this extract, lupulin A (a clerodane diterpenes) exhibited the highest inhibition of COX-1and 6-deoxyharpagide (an iridoid) showed the highest COX-2 inhibition [8]. Cyclooxygenase (COX) catalyzes the biosynthesis of prostaglandin G_2_ and H_2_ from arachidonic acid. The cyclooxygenase isoforms (COX-1 and COX-2) are the target of the non-steroidal anti-inflammatory drugs (NSAIDs), which provide therapeutic effects in the treatment of pain, fever, and inflammation [25].

Another herb that is used in treatment of pain and arthritis is *Harpagophytum procumbens*, commonly known as devil’s claw. The total water extract of this plant possesses anti-inflammatory and analgesic effects by suppressing cyclooxygenase-2 and inducible nitric oxide synthase expressions. Iridoid glycosides such as harpagoside and harpagide are principal constituent of devil’s claw [26,27]. In our previous study, 8-acethyharpagide, an iridoid glycoside, was isolated in a large amount from the diethyl ether fraction [16]. Many studies have exhibited the biological effects of iridoids such as antioxidant, cytotoxic [9], chemoprotective [28], cardiovascular, hypoglycemic and hypolipidemic properties [29]. Li et al. [30] showed that the iridoid glycosides extract of *Lamiophlomis rotate* has a significant antinociceptive effect that was better at the second phase than the first phase of the formalin test [30].

## Conclusion

In conclusion, hexane and diethyl ether fractions obtained from the methanolic extract 80%, and total water extract of aerial parts of *Ajuga chamaecistus* ssp. *tomentella* possess significant and promising antinociceptive properties. The mechanism is supposed to be mediated through the inhibition of endogenous mediators release like prostaglandins. According to the previous study, the isolated compound 8-acethylharpagide could be responsible for the antinociceptive effect of the total water extract and diethyl ether fraction. This study confirmed the traditional use of some *Ajuga* plants for the treatment of joint pain and other inflammatory diseases.

## Competing interests

The authors declare that they have no competing interests.

## Authors' contributions

MK: Participated in introducing the plant and designing the study, AD: carried out the antinociception effect, NS: Participated in extraction of the plant and drafting the manuscript, MRS: Helped to revise the manuscript, MS: Participated in design and interpreting of the data. All authors read and approve the final manuscript.
